# Intravitreal Neuroglobin Mitigates Primate Experimental Glaucomatous Structural Damage in Association with Reduced Optic Nerve Microglial and Complement 3-Astrocyte Activation

**DOI:** 10.3390/biom13060961

**Published:** 2023-06-08

**Authors:** Anita S. Y. Chan, Sai B. B. Tun, Myoe N. Lynn, Candice Ho, Tin A. Tun, Michaël J. A. Girard, Rehena Sultana, Veluchamy A. Barathi, Tin Aung, Makoto Aihara

**Affiliations:** 1 Singapore Eye Research Institute, Singapore 169856, Singapore; sai.bo.bo.tun@seri.com.sg (S.B.B.T.); myoe.naing.lynn@seri.com.sg (M.N.L.); candice.ho.e.h@seri.com.sg (C.H.); tin.aung.tun@seri.com.sg (T.A.T.); mgirard@ophthalmic.engineering (M.J.A.G.); amutha.b.veluchamy@seri.com.sg (V.A.B.); aung.tin@singhealth.com.sg (T.A.); 2 Singapore National Eye Centre, Singapore 168751, Singapore; 3 Duke-NUS Medical School, Singapore 169857, Singapore; rehena.sultana@duke-nus.edu.sg; 4 Ophthalmic Engineering & Innovation Laboratory (OEIL), Singapore Eye Research Institute, Singapore 169856, Singapore; 5 Department of Ophthalmology, Yong Loo Lin School of Medicine, National University of Singapore, Singapore 119077, Singapore; 6 Department of Ophthalmology, University of Tokyo, Tokyo 113-8654, Japan; aihara-tky@umin.net

**Keywords:** glaucomatous optic neuropathy, neuroglobin, complement 3, astrogliosis, microgliosis, neuroprotection

## Abstract

Current management of glaucomatous optic neuropathy is limited to intraocular pressure control. Neuroglobin (Ngb) is an endogenous neuroprotectant expressed in neurons and astrocytes. We recently showed that exogenous intravitreal Ngb reduced inflammatory cytokines and microglial activation in a rodent model of hypoxia. We thus hypothesised that IVT-Ngb may also be neuroprotective in experimental glaucoma (EG) by mitigating optic nerve (ON) astrogliosis and microgliosis as well as structural damage. In this study using a microbead-induced model of EG in six Cynomolgus primates, optical coherence imaging showed that Ngb-treated EG eyes had significantly less thinning of the peripapillary minimum rim width, retinal nerve fibre layer thickness, and ON head cupping than untreated EG eyes. Immunohistochemistry confirmed that ON astrocytes overexpressed Ngb following Ngb treatment. A reduction in complement 3 and cleaved-caspase 3 activated microglia and astrocytes was also noted. Our findings in higher-order primates recapitulate the effects of neuroprotection by Ngb treatment in rodent EG studies and suggest that Ngb may be a potential candidate for glaucoma neuroprotection in humans.

## 1. Introduction

Glaucoma is a neurodegenerative disease that is also one of the most common causes of blindness worldwide [1,2,3]. The hallmarks of glaucoma are progressive retinal ganglion cell loss and glaucomatous optic neuropathy (GON) [1,2,3]. Intraocular pressure (IOP) is the only modifiable risk factor for glaucoma, and thus, the mainstay of glaucoma management is limited to IOP control. To improve glaucoma management, there has been considerable interest in identifying neuroprotective agents that can be used together with IOP-lowering drugs [4,5].

Neuroglobin (Ngb) is a small (17 kDa) hexacoordinated globin that is highly expressed in the brain and retina [6,7]. Ngb was first discovered in 2000 and was described as an endogenous neuroprotectant for neurons [8]. Since then, numerous in vitro studies have demonstrated that Ngb overexpression in both the brain [9,10,11,12,13,14,15] and ocular [16,17,18,19,20,21,22,23] astrocytes and neurons is neuroprotective [9,10,11,12,13,14]. Furthermore, this neuroprotective role of Ngb has also been demonstrated in stroke, retinal ischaemia, and optic nerve disorders in transgenic Ngb-overexpressing rodents in vivo [12,15,16,18,21,23,24]. Direct delivery of Ngb protein [21] or *Ngb* gene therapy [19,21,23,24] in rodent models of retinal hypoxia, glaucoma, and cerebral ischaemia have also confirmed that the neuroprotective effects of Ngb can be reproduced by exogenous delivery.

Studies have suggested that activated astrocytes and microglial cells contribute to optic nerve damage via neuroinflammatory mechanisms [25,26,27,28] involving the release of pro-inflammatory cytokines, such as interleukin (IL)-6, that may exacerbate axonal loss [27,28]. In addition, complement 3 (C3) activated astrocytes have been shown to mediate retinal ganglion cell damage during neuroinflammation [29]. We previously showed that exogenous IVT-Ngb in rodent eyes reduced retinal ganglion cell (RGC) apoptosis by abrogating the production of pro-inflammatory cytokines, including IL-6, and microglial activation during transient hypoxia [21].

Primates, which are phylogenetically similar to humans, are a useful model in clinical translation studies. The primate microbead model of experimental glaucoma (EG) is useful for the investigation of the progressive changes in the optic nerve head (ONH) that are secondary to the IOP risk factor in glaucoma [30,31,32]. Since studies have shown that *Ngb* gene therapy is protective in rodent EG models [16], we hypothesised that intravitreal treatment (IVT) with Ngb (IVT-Ngb) may similarly prevent structural changes to the ONH in primate EG. Furthermore, in optical coherence tomography (OCT), parameters such as the anterior lamina cribrosa depth (LCD), minimum rim width (MRW) thickness, and peripapillary retinal nerve fibre layer (RNFL) thickness [33,34] can be used to monitor the dynamic and progressive structural damage to the ONH in EG [30,35]. Therefore, in this study, we investigated the neuroprotective effects of IVT-Ngb against IOP-mediated structural changes, microgliosis, and astrogliosis in the optic nerve as well as the ocular safety profile of IVT-Ngb using serial OCT measurements in a primate model of EG.

## 2. Materials and Methods

### 2.1. Animal Use and Ethical Approval

All experiments and animal care procedures were performed in an American Association for Accreditation of Laboratory Animal Care (AAALAC) approved animal facility. Procedures were performed in accordance with the Association for Research in Vision and Ophthalmology (ARVO) Statement on the Use of Animals in Ophthalmic and Vision Research and with Institutional Animal Care and Use Committee (IACUC) approval (SingHealth IACUC Code: 2014/SHS/1009).

A total of six Cynomolgus macaque (*Macaca fascicularis*) primates (4 females and 2 males), aged 4–5 years, were used in this study and monitored for a duration of 10 months.

### 2.2. Animal Anaesthesia

All procedures were performed under general anaesthesia (GA) induced by intramuscular injections of a combination of ketamine (15 mg/kg) and medetomidine (0.04–0.008 mg/kg). Topical anaesthesia (1–2 drops of 1% xylocaine) was administered to both eyes before any contact procedures were performed.

### 2.3. EG Induction and IOP Measurements

Using our modified version [30,36] of the previously established primate microbead ocular hypertension (OHT) model [37,38], the IOP was elevated by repeated injections of Dye-Trak (F) fluorescent polystyrene microspheres/microbeads (15 µm diameter) obtained from Triton technology Inc. (San Diego, CA, USA). The microbeads were received at a concentration of 1 million/mL of injectable saline solution containing 0.05% Tween 80 (polysorbate 80, Sigma-Aldrich, St. Louis, MO, USA) and 0.01% Thimerosal. Prior to injection, the microbeads were washed in sterile saline containing 0.001% Tween 80 to remove the Thimerosal and then concentrated to achieve a final concentration of 2 million microbeads/mL. Using a 30-gauge needle mounted on a disposable 1 mL insulin syringe, 50–100 µL of aqueous humour was removed prior to injection of the microbeads (50 µL) into the anterior chamber. The solution was injected slowly to avoid a pressure spike. Re-injection was performed if the IOP was <20 mmHg. OHT was defined as an IOP > 20 mmHg for more than two consecutive readings.

IOP measurements were performed using a TONOVET tonometer (Icare, Finland) at a fixed time in the morning with animals under light anaesthesia (intramuscular ketamine, 5 mg/kg). Five IOP readings were recorded weekly and used to calculate mean monthly IOPs. The mean OHT IOP was calculated once the IOP reached >20 mmHg until the end of study at 10 months.

### 2.4. Optical Coherence Tomography Imaging

**Figure 1:**
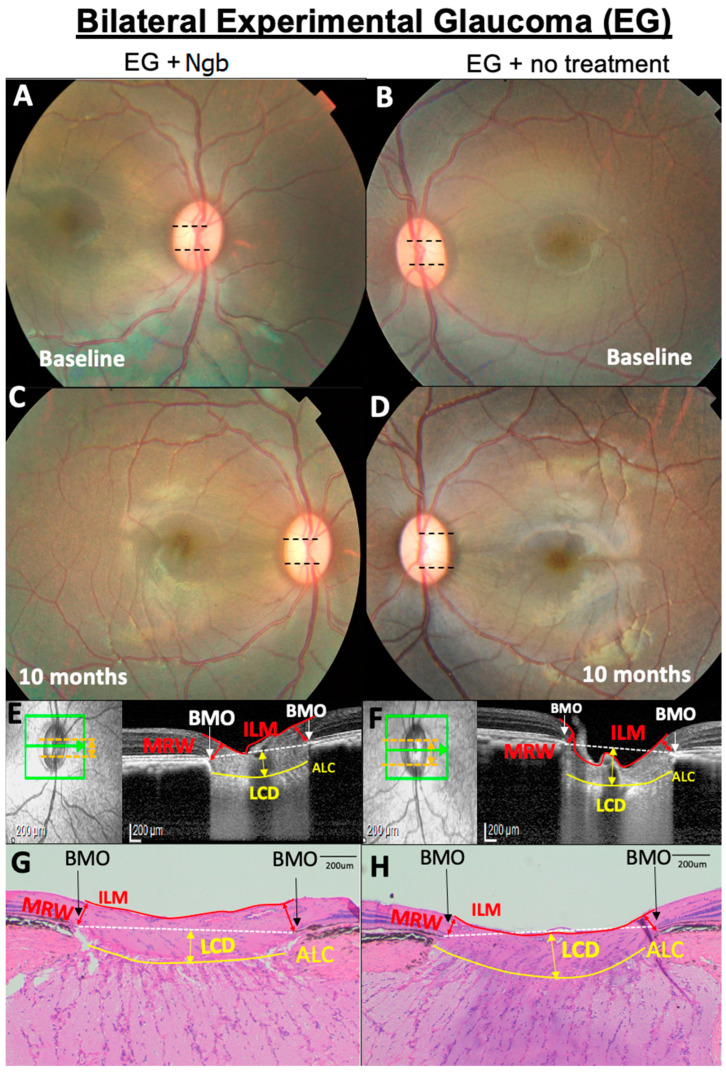
Representative OCT and histology images of the fundus of the optic nerve head (ONH) from primate number 6 showing the optic nerve head (ONH) structural changes. (**A**,**B**) No difference in baseline cup disc ratio (CDR, black dotted lines). (**C**–**H**) At 10 months, CDR in the eye treated with neuroglobin (Ngb) has a smaller CDR (**C**) compared to untreated eyes with EG, which is corroborated by the OCT findings (**E**,**F**) that show increased LCD (yellow arrows) and thinner MRW (red arrows) in the untreated eye (**F**). This is also reflected in the histology of the ONH (**G**,**H**), where there are similar findings of increased LCD and thinner MRW (**H**) in untreated EG eyes. Ngb, neuroglobin; BMO, Bruch’s membrane opening; ILM, internal limiting membrane; MRW, minimum rim width; ALC, anterior lamina cribrosa; and LCD, lamina cribrosa depth.

**Figure 2:**
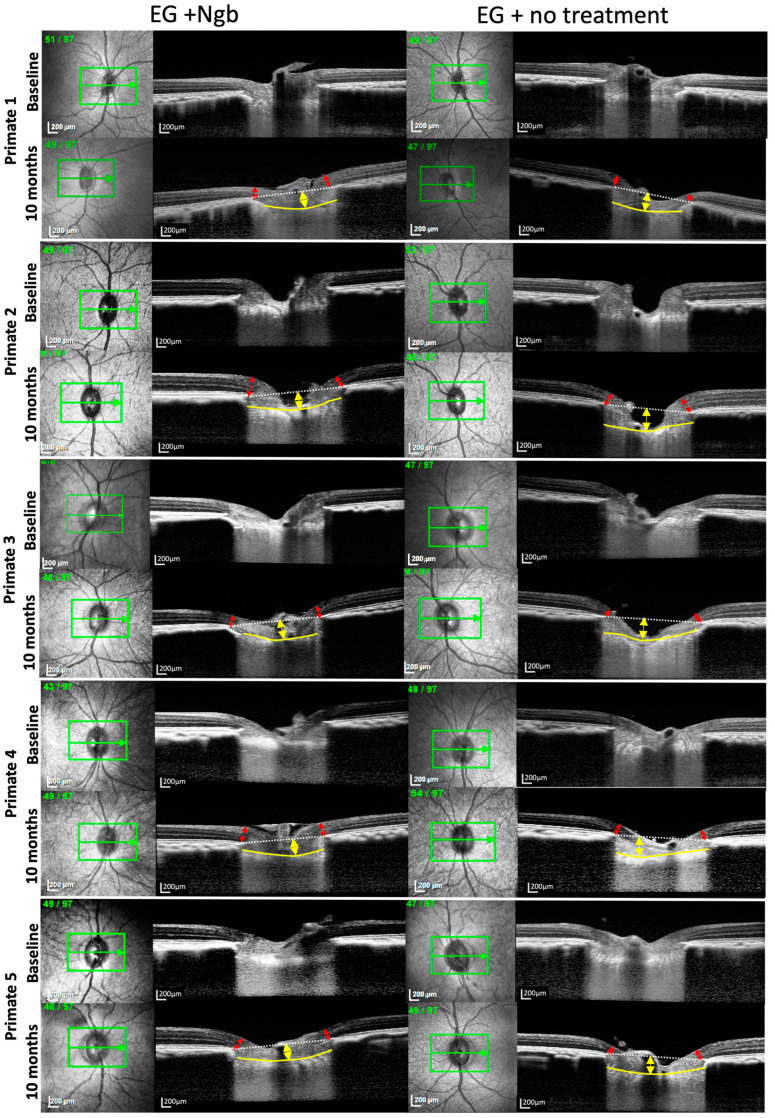
OCT images of the optic nerve head of primates 1–5 also showing reduced LCD deepening and less MRW thinning in IVT-Ngb-treated eyes. Legend: The yellow arrows represent the lamina cribrosa depth (LCD); the red arrows represent the minimal rim width (MRW); the yellow line delineates the anterior lamina cribrosa (LC); and the white dotted line represents the line joining Bruch’s membrane opening (BMO) for calculating the LCD between it and the anterior LC (yellow line). Primate 6 OCT is shown in Figure 1E,F.

Each month during the experimental period, animals were anaesthetised, and spectral domain (SD)-OCT images were acquired on the Spectralis imaging platform (Heidelberg Engineering, Germany) under dim light settings. Each OCT volume consisted of 73 serial horizontal B-scans (62 µm distance between B-scans; 384 A-scans per B-scan) covering a square area of 15° × 15° centred on the ONH. After the volume scan, peripapillary RNFL thickness was acquired using a protocol for RNFL circular OCT scanning around the optic head. The eye-tracking and enhanced depth imaging (EDI) modalities of the Spectralis software were used during image acquisition. Each B-scan was averaged 50 times during acquisition. Segmentation of the post-processed SD-OCT volume of each eye was performed using previously reported, custom-written MATLAB (MathWorks Inc., Natick, MA, USA) algorithms [39,40]. Serial anterior lamina cribrosa depth (LCD), minimum rim width (MRW), and peripapillary RNFL thickness were calculated. The mean RNFL thickness was measured by identifying the internal limiting membrane (ILM) and upper edge of the ganglion cell layer. The LCD was defined as the perpendicular distance from the Bruch’s membrane opening (BMO) reference plane to the anterior lamina cribrosa (LC) surface (Figure 1E,F) [30,39,40]. The anterior LC surface was defined by a sharp increase in axial signal intensity (corresponding to collagen) extending laterally up to the LC insertion points in the peripapillary sclera [30,39,40]. The BMO was defined as the endpoint of Bruch’s membrane layer on either side of the ONH (Figure 1E,F) [39,40]. The anterior LC and BMO were marked manually, while the ILM was delineated automatically by the Spectralis software. The MRW was defined as the shortest distance from the BMO to the ILM (Figure 1E,F and Figure 2) [30,39,40]. To reduce inter-operator errors, all images were acquired by the same individual (SBBT), and all analysis of the IOP, RNFL, LCD, MRW, and RNFL measurements was performed by one individual (MNL). To reduce bias in the interpretation of our findings, all structural measurements were performed in a blinded manner regarding the identity of the Ngb-treated eye.

### 2.5. Anterior Segment Imaging and Fundus Examination

Slit-lamp photography (NS-2D, Righton, Japan) and fundus photography (TRC50Dx, Topcon, Japan) were performed one week after IVT injections and monthly thereafter to assess endophthalmitis, uveitis, and retinal detachment. The cup–disc ratio (CDR) values were also recorded at baseline and over 10 months.

### 2.6. Intravitreal Injections

IVT injections were performed when the IOP was sustained at >20 mmHg, and OCT ONH parameters (LCD, MRW, RNFL) showed evidence of significant ONH changes from baseline. A single IVT dose of Ngb (50 µL at 1 mg/mL; effects of recombinant Ngb protein, CYT-450 (ProSpec-Tany Technogene Ltd., Rehovot, Israel) as shown in our previous study [21]) was injected into the right EG eye of primates in the IVT-Ngb group. The contralateral eyes served as the EG group and received a sham injection of balanced salt solution (BSS).

### 2.7. Histology and Immunohistochemistry

After 10 months, all animals were euthanised, and the globes were enucleated for histological analysis. A rectangular piece of retina–optic nerve complex located 5 mm nasally superiorly and inferiorly, and 10 mm temporal to the ONH, was dissected and formalin-fixed for haematoxylin and eosin (HE) staining. The optic nerve was also transected from the globe and fixed for HE staining and immunohistochemistry analysis. Using previously published histology methods for HE staining, 4-μm thick sections (Microsystems Plus Slides, Leica) were cut and stained using automated HE protocols [27]. Immunohistochemistry (IHC) analysis of the expression of Ngb (Proteintech, 13499-1-AP, 1:50 dilution, Singapore), the astrocyte marker, glial fibrillary acidic protein (GFAP, DAKO, Z0334, 1:6000 dilution), anti-cleaved caspase 3 (Abcam, ab2302, 1:50 dilution), complement 3 (C3, Abcam, ab200999, 1:250 dilution), and the microglial marker, ionised calcium-binding adaptor molecule 1 (IBA1, Abcam, ab178847, 1:800 dilution), was performed using previously published protocols [21]. Dual IHC with GFAP and Ngb, as well as C3 and anti-cleaved caspase 3, was performed to identify astrocytes expressing these proteins.

The mean counts of IBA1-labelled activated microglial cells (identified by the amoeboid appearance) and astrocytes co-labelled with antibodies for the detection of Ngb protein, C3, and cleaved-caspase 3, were quantified manually from three consecutive sections within 5 mm of the ONH in the EG (sham-treated) and IVT-Ngb-treated groups.

### 2.8. Statistical Analyses

A repeated-measures analysis of variance was used with time (baseline, follow-up of 10 visits, every month) and EG and IVT-Ngb-treated eyes as factors. Differences in OCT parameters (LCD, MRW, and RNFL) and cell counts between EG and IVT-Ngb-treated eyes were evaluated using a repeated measure *t*-test. *p* < 0.05 was set as the threshold for statistical significance. Analysis was carried out using SAS9.4.

## 3. Results

### 3.1. Baseline Parameters before EG Induction Are Similar

The mean baseline values for IOP, CDR, LCD, MRW, and RNFL are reported in Table 1. There was no significant difference in the baseline values for the IOP (*p* = 0.611), CDR (*p* = 0.687), LCD (*p* = 0.890), MRW (*p* = 0.628), and RNFL (*p* = 0.385) in both the right and left eyes, indicating a similar degree of EG induced in both eyes and, thus, confirming that any subsequent changes in OCT were not due to asymmetric EG induction. Baseline fundus imaging (Figure 1A,B) showed no evidence of uveitis, and all primates had normal CDRs (0.2 ± 0.1, Table 1).

### 3.2. IOP Profiles from Six Primates Show Similar IOP Elevations

A mean of 5 ± 1 intracameral microbead injections were required to achieve ocular hypertension (OHT) in both eyes. Bilateral IOP elevation was achieved by month (M) 2 (M2, Table 2, IOP profile). There was no significant difference in the mean IOP of the right and left eyes between M2 and M10, which was the period of OHT in the EG model (Table 2 IOP profile). Similarly, there was no significant difference in the mean maximal IOP of the left eye (53.0 ± 15.9 mmHg) and the right eye (55.0 ± 8.7 mmHg) (Table 2). In addition, there was no significant difference in the mean change in the IOP from baseline in the left eye (10.1 ± 6.67 mmHg) and in the right (Ngb-treated) eye (14.6 ± 6.6 mmHg) (Table 2 IOP profile). Since changes in IOP can have structural impacts [31], the absence of difference between the mean IOP, mean change in IOP, and mean maximal IOP between the two eyes reduces the potential confounding effects on the outcome due to IOP differences.

### 3.3. OCT Structural Changes with EG: Before and after Intervention

**Figure 3:**
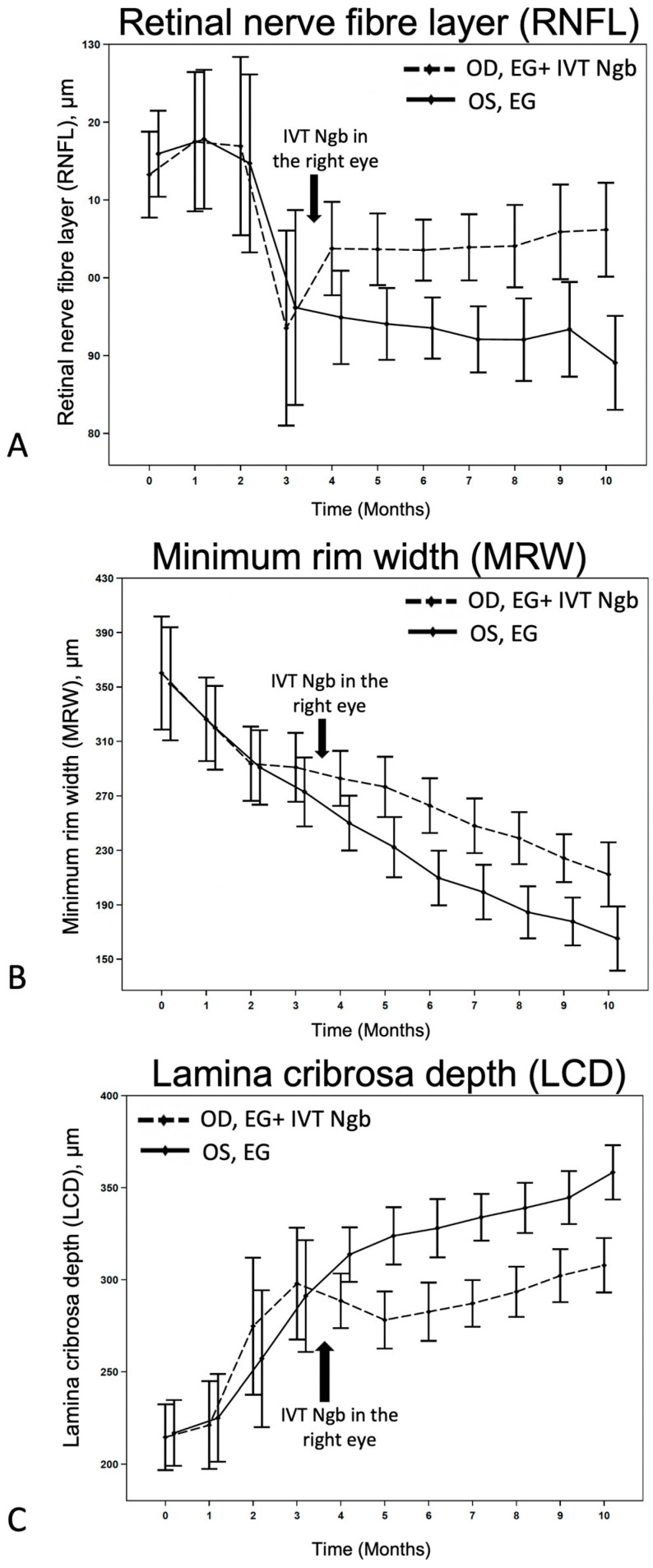
Effects of IVT-Ngb on OCT measurements in bilateral EG. (**A**–**C**) The effects of IVT-Ngb (OD) and IVT-Sham (OS) injections performed in the right and left eyes, respectively, at 3.5 months on subsequent RNFL (**A**), MRW (**B**), and LCD (**C**) measured by OCT from months 4 to 10. Data represent the mean ± standard deviation. Please refer to Table 3 for *p*-values at monthly time points.

OCT images of primates 1–5 and 6 are presented in Figure 1E,F and Figure 2, respectively. Serial MRW and LCD depth measurements from baseline to M10 are summarised in Figure 3 and Table 3.

We first monitored the OCT changes in the ONH due to EG before intervention. During M2–M3 of initial ocular hypertension (Table 2), early changes were seen in the RNFL, LCD, and to a lesser degree, in the MRW (Figure 3), although these changes were not statistically significant (Table 3). This finding confirmed that the initial IOP elevation was associated with early evidence of structural change and indicated the ideal timing for intervention.

We then investigated the effects of IVT-Ngb (OD) and IVT-sham (OS) injections performed in the right and left eyes, respectively, at 3.5 months on subsequent OCT measurements. At the initial post-intervention OCT analysis at M4, there were significant changes in the mean RNFL, LCD, and MRW between the right (OD) IVT Ngb-treated eyes and the left (OS) untreated eyes (Table 3, M4: RNFL, *p* = 0.0417; LCD, *p* = 0.0194; MRW, *p* = 0.0246). The RNFL, LCD, and MRW OCT parameters continued to show statistically significant differences between both eyes from M5 to the study endpoint at M10, despite no further intervention and chronic ocular hypertension (Table 3, Figure 3). At the end of the study, the percentage change from M2 to M10 (period of ocular hypertension) between both eyes showed that the RNFL of the untreated left eyes with EG was significantly thinner than the RNFL of the IVT-Ngb-treated eyes with EG (*p* = 0.0047), suggestive of a greater loss in RGC. Similarly, the percentage changes in LCD from M2 to M10 between both eyes also showed that IVT-Ngb-treated eyes with EG had significantly less deepening of the LCD than untreated eyes with EG (*p* = 0.0064). Although there was no significant difference in the percentage change in the MRW between the right (treated) and left (untreated) eyes between M2 and M10 (Table 3, MRW), which suggests that the rate of change for MRW is similar, it is important to note that the MRW showed significant monthly decreases in thinning in the IVT-Ngb-treated eyes. This similar rate of change may be accounted for by the reported greater sensitivity of MRW in detecting structural change than RNFL and LCD.

### 3.4. IVT-Ngb Increased the Cup–Disc Ratio after EG

The mean CDR in EG eyes at M10 was significantly increased from the baseline CDR (0.4 ± 0.1 vs. 0.2 ± 0.1, *p* = 0.001). In contrast, there was no significant difference in the mean CDR of IVT-Ngb-treated eyes at M10 0.3 ± 0.1 compared with the baseline CDR (0.3 ± 0.1 vs. 0.2 ± 0.1, *p* = 0.541).

### 3.5. Histological Changes in the ONH Corroborate OCT Structural Changes

Histological evaluation of the ONH and peripapillary retina at M10 (Figure 1G,H) confirmed the presence of clinical cupping. HE staining showed a mild increase in LCD and increased peripapillary RNFL and MRW thinning in the EG eyes (Figure 1H) compared to the corresponding section in the IVT-Ngb-treated eyes (Figure 1G) and were consistent with the in vivo OCT images. No infiltration by inflammatory cells such as lymphocytes and neutrophils was observed.

### 3.6. IVT-Ngb Increases Ngb Expression in the Optic Nerve and Reduces Infiltration by Activated Astrocytes and Microglial Cells

**Figure 4:**
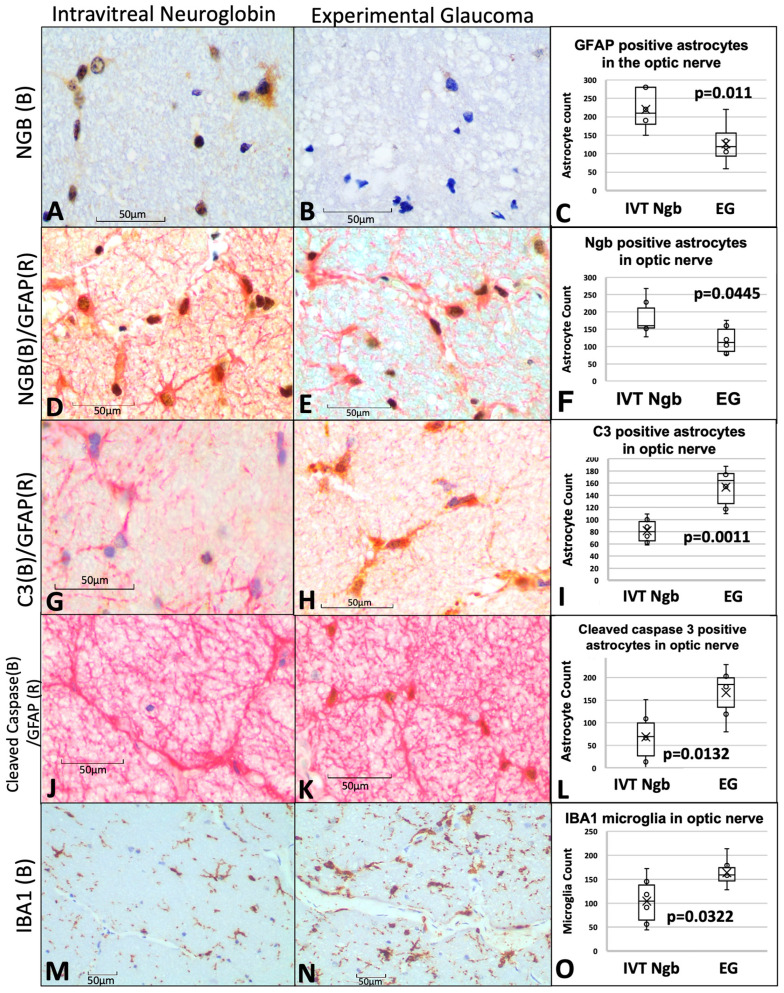
Optic nerve immunohistochemistry and cell counts of astrocytes, microglial, and neuroglobin expression. (**A**–**C**) Immunohistochemistry staining of Ngb (brown chromogen) in IVT-Ngb-treated eyes (**A**) and IVT-sham-treated eyes (**B**) in a primate model of experimental glaucoma (EG). Cell counts of co-stained Ngb-positive astrocytes (with GFAP, **D**,**E**). (**D**–**F**) Dual staining of the astrocyte markers, GFAP (red chromogen) and Ngb (brown chromogen) in IVT-Ngb-treated eyes (**D**) and IVT-sham-treated eyes (**E**,**F**). (**G**–**I**) Complement 3 (C3, brown chromogen) expression in the GFAP-positive astrocytes of IVT-sham-treated eyes (**H**) and IVT-Ngb eyes (**G**). (**J**–**L**) Cleaved-caspase 3 (brown chromogen) expression in the GFAP-positive astrocytes of IVT-sham-treated eyes (**K**) and IVT-Ngb eyes (**J**). (**M**–**O**) Expression of the microglial marker, IBA1 (brown chromogen), increased in IVT-sham-treated eyes (**N**,**O**) and IVT-Ngb eyes (**M**). Data represent mean ± standard deviation (*t*-test). LEGEND: IVT, intravitreal; Ngb, neuroglobin; EG, experimental glaucoma; (B), brown; (R), red; GFAP, glial fibrillary acid phosphatase; C3, complement 3; and IBA1, ionised calcium-binding adapter molecule 1.

Ngb expression was increased in the IVT-Ngb-treated optic nerves (Figure 4A) in comparison to Ngb expression in the EG optic nerves (Figure 4B). This increased Ngb expression was found to co-localise with GFAP-labelled (GFP+) astrocytes (Figure 4D,E). There was a significantly higher number of astrocytes positive for Ngb (Ngb+) in the IVT-Ngb-treated optic nerves (mean NGB+ GFAP+ count, 148.40 ± 51.32 SD, Figure 4D–F) than those in EG untreated optic nerves (mean NGB+ GFAP+ count, 119.33 ± 41.29 SD, *p* = 0.0445 Figure 4D–F), suggesting that the IVT-Ngb injection increased Ngb expression in astrocyte.

Overall mean counts of GFAP+ astrocytes cells were significantly elevated in the optic nerves of IVT-NGB eyes (mean GFAP+ astrocytes, 220.00 ± 51.77) in comparison to EG eyes (mean GFAP+ astrocytes, 126.17.50 ± 52.81, *p* = 0.011, Figure 4C).

To determine the proportion of these astrocytes that are activated, we co-localised C3 and cleaved-caspase 3 expressions as markers of astrocyte activation. We found a significantly decreased mean number of C3+ astrocytes (152.39 ± 32.70 vs. 81.83 ± 20.63, *p* = 0.0011, Figure 4G–I) and cleaved-caspase 3+ astrocytes (200.67 ± 100.64 SD vs. 87.17 ± 67.03, *p* = 0.003, Figure 4J–L) in EG eyes when compared with IVT-Ngb-treated eyes. This suggests that there is a greater proportion of reactive pro-inflammatory astrocytes in EG.

A significantly greater number of activated microglial cells with an amoeboid shape (microgliosis) was also detected in the optic nerves of EG eyes (mean IBA1 microglial cell count 163.67 ± 32.55) in comparison to IVT-Ngb-treated eyes (mean IBA1 microglial cell count 104.33 ± 51.63, *p* = 0.003, Figure 4M–O).

In summary, IVT-Ngb appeared to increase Ngb expression in astrocytes and was associated with a reduction in activated astrocytes that expressed C3 and cleaved-caspase 3. This reduction in activated astrocytes is associated with decreased OCT structural and ONH CDR progression.

### 3.7. Complications Post-IVT Injection included a Bilateral Transient Uveitis

One week post-IVT injections, both the IVT-Ngb and EG sham-injected eyes showed transient mild-to-moderate anterior and posterior segment inflammation. This resolved within two weeks after the application of topical corticosteroids to both eyes for the same period. No vasculitis or retinitis was noted when the inflammation was resolved, and there was no persistent uveitis at the end of the study (Figure 1C,D). There was no evidence of infection, cataracts, or retinal detachment post-IVT injections at the end of the study (Figure 1C,D).

## 4. Discussion

In this study, we investigated the effects of exogenous IVT-Ngb in a primate model of EG by studying the IOP-mediated structural changes based on OCT measurements as well as the astroglia and microglia of the optic nerve. We also evaluated the ocular safety profile of IVT-Ngb.

We recently reported the ONH OCT changes induced by the microbead approach to IOP elevation in primates and, thus, used this primate model of EG and similar OCT protocols for this study [30]. As different animals may vary in their susceptibility to IOP damage, we chose a bilateral rather than the unilateral EG model to minimise inter-animal differences. Using this model, we previously reported that both the mean and maximum IOP were significant predictors of changes in the MRW and LCD [30]. In this study, there was no significant difference between these two IOP parameters in both eyes (Table 2), indicating that the IOP insult, which served as the main risk factor for glaucoma development in our study, was similar in both the IVT-Ngb-treated and EG (sham-treated) eyes.

Inter-eye asymmetry of structural change has been reported in bilateral glaucoma [41]; therefore, to avoid this confounding factor, baseline and serial OCT MRW, LCD, and RNFL parameters were compared prior to IVT-Ngb treatment. We chose to perform the IVT-Ngb injections when the initial changes in MRW, LCD, and RNFL did not show significant changes between the eyes (M2, M3) after the elevation of the IOP at M2 (Table 1 and Table 3). With these potential confounders minimised, we can then be confident that the structural changes seen in this study can be attributed mainly to IVT-Ngb treatment rather than variations in IOP and susceptibility to damage.

After treatment at M3.5, IVT-Ngb-treated eyes showed significantly less structural damage in all three OCT parameters (LCD, MRW, and RNFL) as early as M4 compared to the EG (sham-treated) eyes (all *p* < 0.05, Table 3, Figure 3). This trend of reduced structural changes (reduced deepening of LCD and decreased thinning of MRW and RNFL) in IVT-Ngb-treated eyes compared to EG (sham-treated) eyes continued each month up to M10 (end of study). Together with ex vivo optic nerve histology that confirmed the in vivo OCT changes, these findings suggest that Ngb is neuroprotective against structural damage.

In the brain and optic nerve, astrocytes and microglial cells are known to play important roles in neuronal homeostasis [27,28]. Recent studies suggest that astrocytes around the ONH can be activated and become reactive (reactive astrogliosis) in the early stages of IOP elevation, but may decrease together with axonal loss and long-term IOP elevation [42,43,44]. In addition, these studies suggest that reactive astrogliosis in the early stages of glaucoma may also be neuroprotective [42,43]. Reactive astrocytes (A) can be divided into A1 and A2 subtypes [43,45,46], although this subclassification is not strictly binary. A2 astrocytes are usually reported to be neuroprotective, while A1 astrocytes are associated with a more neurotoxic phenotype [46]. A1 astrocytes may express C3 and show nuclear expression of cleaved-caspase 3 [46,47,48]. Although caspase 3 is an executor of death proteases and a known mediator of apoptosis, studies on stroke and post-excitotoxic damage in the brain have shown that nuclear expression of cleaved-caspase 3 in activated A1 astrocytes may also contribute to microglial and astrocyte activation [47,48]. Similarly, microgliosis is known to be an early response to IOP elevation and has been suggested as a predictor of neurodegeneration in glaucoma [44,49,50].

In this study, Ngb overexpression occurred specifically in GFAP-labelled astrocytes (Figure 4D–F), which is consistent with studies that have shown its expression in astrocytes, in addition to neurons [51,52]. In the untreated eyes with EG, there was a significantly higher number of reactive A1 neurotoxic phenotype astrocytes characterised by the expression of C3 and cleaved-caspase 3 at M10 (Figure 4I,L). This feature was associated with significantly increased OCT evidence of LCD deepening and RNFL thinning (Table 3) compared to Ngb-treated eyes with EG. Furthermore, these reactive astrocytes also lacked Ngb expression (Figure 4C). Overall, there were significantly fewer astrocytes in untreated ONH (Figure 4F) compared to IVT-Ngb ONH, which is consistent with the astrocyte loss in the ONH of mice with chronic ocular hypertension [42,49,50]. This change was also associated with increased reactive IBA1a expression in the microglia (Figure 4O).

In contrast, in the IVT-Ngb-treated eye with EG, the ONH had significantly greater numbers of NGB+/GFAP+ astrocytes with reduced expression of C3 and cleaved-caspase 3 (the reactive or neurotoxic phenotype, Figure 4I,L). The significantly less marked changes in CDR from baseline and reduced OCT structural changes in these eyes suggest that Ngb overexpression is one of the factors that may mitigate the structural changes induced by EG by reducing the number of reactive astrocytes, leading to the overall preservation of astrocytes. The lack of C3 and cleaved-caspase 3 in some of these astrocytes suggests that they may be of the A2 neuroprotective phenotype and warrants further investigation. This observation is further supported by the smaller numbers of reactive IBA1a microglia expression in Ngb-treated eyes (Figure 4O) and is also consistent with our previous report that retinal Ngb overexpression reduced the retinal neuroinflammatory response in a rat model of transient hypoxia [21].

The limitations of our study should be noted. First, this study was performed on a small sample size of only six primates, which was limited due to ethical and cost constraints. Nevertheless, the sample size was similar to that in other primate studies, which averaged between 6 and 8 primates [53]. In addition, studies with 3–8 primates have been shown to be adequately powered to detect differences between groups [53]. Thus, a larger series of studies incorporating extensive molecular investigations are required to confirm our findings. Second, we encountered transient inflammation in both eyes after the IVT injection. As this complication also occurred in the IVT-sham eyes, we treated both eyes with topical corticosteroids for the same period to minimise the effects of inflammation and corticosteroid therapy on the study outcomes. The use of topical steroid therapy did not seem to affect the neuroinflammatory response seen in EG. However, this minor complication emphasises the need for strict sterility and care in the preparation of drugs for IVT therapy, where any form of contamination can induce inflammation or even endophthalmitis. It should be noted that there were no cases of endophthalmitis in any of the primates in this study. Third, we did not perform dose adjustment or pharmacokinetic analysis, which are important to determine the ideal dose for response and determine the toxic dose limit. However, as this is a pilot study, we used the adjusted dose from our rodent study as the baseline, taking into consideration the larger primate eye volume. Finally, we acknowledge that we did not perform in vitro evaluations to confirm the direct molecular associations between Ngb and astrogliosis. However, expression of Ngb has been reported in astrocytes in vitro as well as in murine models of traumatic brain injury and other brain pathologies [51,52,54].

**Figure 5:**
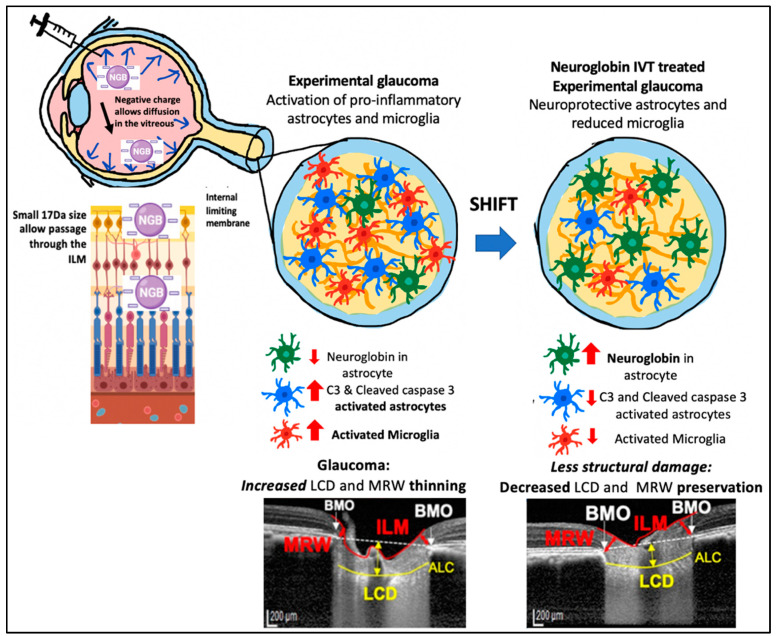
Potential mechanism of action for IVT Ngb mediated reduced pro-inflammatory astrocytes and microglia leading to reduced structural damage. Negatively charged Ngb molecules diffuse through the vitreous. Its small (17 kDa) size allows it to pass the internal limiting membrane (ILM) barrier to reach the optic nerve head (ONH). Its action on astrocytes results in a shift from pro-inflammatory astrocytes to “neuroprotective” phenotype. This leads to decreased structural damage (preservation of MRW thickness and decreased lamina cribrosa depth).

Based on our findings and other reports, we propose the following mechanism by which IVT-Ngb may penetrate the retina and increase Ngb expression in astrocytes. First, the negative charge of the Ngb protein facilitates its diffusion through the vitreous to reach the retina and ONH [55]. The small size of the Ngb protein (17 kDa size) also allows it to penetrate the internal limiting membrane of the retina to reach the RGC and retinal and optic nerve head astrocytes [56]. In GON, ONH astrocytes have been reported to precede RGC loss during early glaucoma insults. Studies suggest that the mechanical IOP and/or ischaemic factors at the ONH lead to the activation of pro-inflammatory astrocytes, which further activates a cascade of oxidative stress factors, cytokines, and caspase 3 activation that leads to RGC loss [43,44,50,57]. In vitro studies show that Ngb treatment of cultured mouse astrocytes reduces reactive oxygen species production and pro-inflammatory cytokine (IL6) release. This results in reduced mitochondrial oxidative stress and decreased caspase 3 activation [51]. In our study, IVT-Ngb increases optic nerve Ngb expression in the astrocytes, which results in decreased levels of activated C3 and caspase 3 expression in optic nerve astrocytes, suggesting there is a shift towards the neuroprotective astrocyte phenotype in comparison to the pro-inflammatory astrocytes seen in the untreated EG optic nerves. This, in turn, is associated with the decreased structural damage in the eyes of EG model primates treated with Ngb (Figure 5, summary of proposed mechanism).

## 5. Conclusions

Despite adequate IOP control, some patients continue to develop worsening optic neuropathy, and thus, there is a need for a non-IOP-mediated neuroprotective therapy [4]. This study suggests that IVT-Ngb therapy increases Ngb expression in astrocytes and is associated with a decreased proportion of reactive neurotoxic astrogliosis that expresses C3 and activated caspase 3 as well as microglial activation, which ameliorates the ONH changes during primate EG when compared to those observed in untreated primates. Although neuroprotective therapies are not currently available for glaucoma patients, the findings of our study in a higher-order animal model, together with previous reports of rodent studies showing similar effects of Ngb in EG, may pave the way for the development of Ngb as a potential neuroprotective therapeutic agent in clinical glaucoma.

## Figures and Tables

**Table 1:** Baseline demographics of the six primates.

		Baseline for IVT-Ngb (Right) Eyes				Baseline for EG (Left) Eyes				
ID	IOP, mmHg	CDR	LCD, µm	MRW, µm	RNFL, µm	IOP, mmHg	CDR	LCD, µm	MRW, µm	RNFL, µm
1	13.0 ± 1.2	0.1	200.7 ± 29.3	292.6 ± 43.6	108.9 ± 42.7	15.0 ± 1.7	0.1	192.1 ± 21.3	264.3 ± 52.9	108.9 ± 42.7
2	16.0 ± 1.7	0.2	213.7 ± 33.5	293.4 ± 57.3	118.6 ± 43.6	15.0 ± 2.3	0.2	223.6 ± 31.2	291.0 ± 53.1	118.6 ± 43.6
3	14.0 ± 1.9	0.3	236.4 ± 6.7	304.7 ± 57.4	119.7 ± 42.9	12.0 ± 1.7	0.3	221.2 ± 14.1	289.0 ± 55.3	119.7 ± 42.9
4	15.0 ± 0.5	0.2	221.4 ± 21.4	277.0 ± 47.6	110.0 ± 36.2	14.0 ± 0.6	0.2	225.4 ± 21.2	277.5 ± 52.4	110. ± 36.2
5	16.0 ± 2.9	0.3	193.0 ± 33.6	341.1 ± 66.6	122.6 ± 44.4	17.0 ± 2.1	0.2	178.1 ± 32.1	360.7 ± 62.3	122.6 ± 44.4
6	12.0 ± 2.3	0.2	197.7 ± 9.2	366.2 ± 46.8	115.9 ± 44.7	16.0 ± 1.0	0.2	217.4 ± 4.7	372.7 ± 46.4	115.9 ± 44.7
Mean	14.3 ± 1.6	0.2 ± 0.1	210.5 ± 16.5	312.7 ± 34.2	115.9 ± 5.5	14.8 ± 1.7	0.2 ± 0.1	209.6 ± 19.7	309.2 ± 45.7	113.2 ± 4.7

Baseline IOP (right eye vs. left eye), *p* = 0.611; baseline CDR (right eye vs. left eye), *p* = 0.687; baseline LCD (right eye vs. left eye), *p* = 0.858; baseline MRW (right eye vs. left eye), *p* = 0.7909; and baseline RNFL (right eye vs. left eye), *p* = 0.496. There was no statistically significant difference in baseline parameters. ID, primate identification number; IOP, intraocular pressure; CDR, cup–disc ratio; LCD, lamina cribrosa depth; MRW, minimum rim width; and RNFL, retinal nerve fibre layer.

**Table 2:** Mean monthly IOP profile, as well as mean and maximum IOP measurements during the hypertensive phase (M2–M10) of experimental glaucoma in each primate.

Individual Animal	Baseline IOP	M1 IOP	M2 IOP	M3 IOP	M4 IOP	M5 IOP	M6 IOP	M7 IOP	M8 IOP	M9 IOP	M10 IOP	Max IOP	Mean IOP M2–10
Primate 1 OD (Ngb)	13	15	45	30	33	42	29	24	27	39	30	45	33
Primate 1 OS	15	13	20	25	28	25	23	36	47	40	32	47	31
Primate 2 OD (Ngb)	16	13	23	28	38	43	50	32	50	57	20	57	38
Primate 2 OS	12	15	66	56	35	27	27	36	46	56	31	66	42
Primate 3 OD (Ngb)	14	23	41	38	43	38	15	50	12	20	18	50	31
Primate 3 OS	14	15	25	44	43	28	21	26	22	33	23	44	29
Primate 4 OD (Ngb)	15	15	23	49	40	33	27	19	42	56	21	56	34
Primate 4 OS	17	15	54	24	40	77	56	34	33	45	23	77	43
Primate 5 OD (Ngb)	16	16	56	25	56	28	26	23	54	70	24	70	40
Primate 5 OS	15	11	41	26	28	29	23	26	27	50	30	50	31
Primate 6 OD (Ngb)	12	12	32	50	26	45	20	23	21	24	19	50	29
Primate 6 OS	16	18	27	31	30	32	22	29	21	33	25	33	28
All animals	Monthly mean intraocular pressure in all six animals											Mean Max IOP	Change in IOP M2–10
	Baseline	M1	M2	M3	M4	M5	M6	M7	M8	M9	M10		
Left eye, OS	14.6 ± 5.1	14.4 ± 5.1	33.1 ± 5.1	26.7 ± 5.1	28.8 ± 5.1	39 ± 5.1	20.6 ± 5.4	29.5 ± 5.1	25.5 ± 5.1	22.1 ± 5.1	23 ± 5.1	53 ± 15.9	10.1 ± 6.6
Right eye, OD (Ngb)	15.7 ± 5.1	15.8 ± 5.1	33.8 ± 5.1	26.3 ± 5.1	42.9 ± 5.1	32.4 ± 5.1	27.3 ± 5.4	28.4 ± 5.1	26.6 ± 5.1	21.8 ± 5.1	19.2 ± 5.1	55 ± 8.7	14.6 ± 6.6
*p*-value (OD between OS)	0.611	0.543	0.818	0.742	0.301	0.836	0.912	0.607	0.901	0.871	0.056	0.810	0.127

IOP profile at each time point shows no significant difference (*p* > 0.05). There was no significant difference in mean Max IOP between the right and left eyes, *p* = 0.810). No significant difference in mean change in IOP between the right and left eye, *p* = 0.127. M, month; IOP, intraocular pressure; OS, left eye; OD, right eye; M, month; Max, maximum; and Ngb, neuroglobin.

**Table 3:** OCT measurements in bilateral EG with IVT neuroglobin (Ngb) treatment (right eye) and no treatment (left eye).

Time-Point	RNFL, µm				LCD, µm				MRW, µm			
	OS	OD IVT	Difference (95%CI)	*p*-Value	OS	OD IVT	Difference (95%CI)	*p*-Value	OS	OD IVT	Difference (95%CI)	*p*-Value
Baseline	115.93 (2.78)	113.24 (2.78)	2.69 (−5.12, 10.5)	0.4961	216.94 (9.00)	214.65 (9.00)	2.29 (−22.97, 27.55)	0.8576	352.38 (20.94)	360.25 (20.94)	−7.87 (−66.63, 50.88)	0.7909
M1	117.79 (4.5)	117.47 (4.5)	0.32 (−12.32, 12.95)	0.9604	225.07 (11.98)	221.22 (11.98)	3.86 (−29.75, 37.46)	0.8204	320.07 (15.49)	326.27 (15.49)	−6.20 (−49.67, 37.27)	0.7778
M2	114.69 (5.76)	116.91 (5.76)	−2.22 (−18.39, 13.96)	0.7864	257.18 (18.70)	274.78 (18.7)	−17.61 (−70.08, 34.87)	0.5071	290.92 (13.78)	293.6 (13.78)	−2.68 (−41.35, 35.98)	0.8907
M3	96.17 (6.31)	93.52 (6.31)	2.65 (−15.06, 20.35)	0.7675	291.11 (15.28)	297.88 (15.28)	−6.78 (−49.67, 36.11)	0.7546	272.86 (12.75)	290.98 (12.75)	−18.13 (−53.9, 17.64)	0.3171
M4	94.91 (3.03)	103.75 (3.03)	−8.84 (−17.34, −0.34)	0.0417	313.64 (7.47)	288.53 (7.47)	25.11 (4.15, 46.07)	0.0194	249.99 (10.18)	282.84 (10.18)	−32.84 (−61.4, −4.28)	0.0246
M5	94.06 (2.33)	103.65 (2.33)	−9.59 (−16.12, −3.06)	0.0044	323.79 (7.83)	278.12 (7.83)	45.67 (23.7, 67.64)	<0.0001	232.3 (11.15)	276.63 (11.15)	−44.33 (−75.61, −13.04)	0.0059
M6	93.54 (1.98)	103.55 (1.98)	−10.01 (−15.57, −4.46)	0.0005	327.92 (7.99)	282.57 (7.99)	45.35 (22.94, 67.75)	0.0001	209.64 (10.12)	262.86 (10.12)	−53.22 (−81.62, −24.81)	0.0003
M7	92.09 (2.14)	103.91 (2.14)	−11.82 (−17.83, −5.81)	0.0002	333.87 (6.39)	287.1 (6.39)	46.76 (28.82, 64.71)	<0.0001	199.3 (10.11)	248.01 (10.11)	−48.71 (−77.09, −20.33)	0.0010
M8	92.04 (2.67)	104.07 (2.67)	−12.02 (−19.51, −4.53)	0.0019	338.94 (6.86)	293.4 (6.86)	45.54 (26.3, 64.79)	<0.0001	184.48 (9.65)	238.87 (9.65)	−54.40 (−81.47, −27.33)	0.0001
M9	93.37 (3.07)	105.89 (3.07)	−12.53 (−21.13, −3.92)	0.0048	344.55 (7.25)	302.16 (7.25)	42.39 (22.04, 62.74)	<0.0001	177.66 (8.87)	224.25 (8.87)	−46.59 (−71.49, −21.69)	0.0003
M10	89.07 (3.04)	106.17 (3.04)	−17.09 (−25.63, −8.56)	0.0001	358.27 (7.45)	307.78 (7.45)	50.49 (29.59, 71.38)	<0.0001	165.16 (11.89)	212.27 (11.89)	−47.11 (−80.49, −13.74)	0.0061
Percentage change at M10 from M2	−23.04 (9.22)	−6.10 (5.85)	−16.94 (−27.12, −6.76)	0.0047	65.31 (11.59)	43.85 (9.85)	21.46 (7.57, 35.35)	0.0064	−52.84 (11.18)	−40.81 (9.78)	−12.03 (−25.58, 1.51)	0.0759

## Data Availability

All data generated or analysed during this study are included in this published article. The datasets used and/or analysed during the current study are also available from the corresponding author upon reasonable request.

## Abbreviations

IOP	Intraocular pressure
Ngb	Neuroglobin
ONH	Optic nerve head
OCT	Optical coherence tomography
LCD	Lamina cribrosa depth
MRW	Minimum rim width
RNFL	Retinal nerve fibre layer
EG	Experimental glaucoma
IVT	Intravitreal treatment
IVT-Ngb	Intravitreal treatment with neuroglobin
RGC	Retinal ganglion cell
IL	Interleukin
C3	Complement 3
AAALAC	American Association for Accreditation of Laboratory Animal Care
ARVO	Association for Research in Vision and Ophthalmology
IACUC	Institutional Animal Care and Use Committee
GA	General anaesthesia
OHT	Ocular hypertension
SD	Spectral domain
EDI	Enhanced depth imaging
LC	Lamina cribrosa
BMO	Bruch’s membrane opening
ILM	Internal limiting membrane
CDR	Cup–disc ratio
HE	Haematoxylin and eosin
IHC	Immunohistochemistry
GFAP	Glial fibrillary acid phosphatase
IBA1	Ionised calcium-binding adapter molecule 1
GFAP+	GFAP-positive
Ngb+	Ngb-positive
C3+	Complement 3-positive
M	Month
A	Reactive astrocytes
